# Perceived socially responsible HRM, employee organizational identification, and job performance: the moderating effect of perceived organizational response to a global crisis

**DOI:** 10.1016/j.heliyon.2022.e11563

**Published:** 2022-11-16

**Authors:** Thinh-Van Vu

**Affiliations:** Department of Human Resource Management, Thuongmai University, Hanoi, Vietnam

**Keywords:** Socially responsible HRM, Sustainable HRM, Employees organizational identification, Organizational response to a crisis, COVID-19, Job performance

## Abstract

In an uncertain economy and a globalized world, socially responsible human resource management (HRM) is pivotal to the long-term growth of organizations. This research employed social exchange theory and social identity theory to analyze the correlations between employees' perceptions of socially responsible HRM, organizational identification, and job performance. This research also explored the moderating effect of employees' perceptions of their organization's response to a global crisis such as the COVID-19 pandemic on the relationship between organizational identification and job performance. Analyzing the survey data from 367 respondents using partial least squares structural equation modeling (PLS-SEM) with SmartPLS 3.2 software, this study found that HRM that is perceived to be socially responsible positively influences organizational identification and job performance. Moreover, the study found that organizational identification serves as a mediator between socially responsible HRM and work performance. It also revealed that perceived organizational response to a crisis such as the COVID-19 pandemic positively influences employees' job performance and negatively moderates the nexus between organizational identification and job performance. This study clarified the role of socially responsible HRM and organizational reactions to a crisis in promoting employee job performance.

## 1 Introduction

Socially responsible human resource management (HRM) practices are adopted to nurture employee attitudes and behaviors to improve the performance of organizations’ internal and external social responsibility initiatives (Luu, 2021; Zhang et al., 2022). There have been many definitions of socially responsible HRM, which have all converged on the combination of corporate social responsibility (CSR) and sustainable HRM (Shen and Benson, 2016). Socially responsible HRM can raise awareness of the effect of a business on employees beyond organizational boundaries and time frames. Moreover, the implementation of socially responsible HRM could result in a more ethical and sustainable workplace (Kundu and Gahlawat, 2015). According to the findings of Del-Castillo-Feito et al. (2022), implementing socially responsible HRM also plays a crucial role in achieving social acceptance. Consequently, socially responsible HRM helps accomplish organizational consistency with a CSR framework for the sustainable development of organizations.

According to Zhang et al. (2022), there is a potential research gap in the theoretical knowledge of the social impact of socially responsible HRM practices. The literature review also indicates that few studies have explored the influence of employees' perceived socially responsible HRM on individual employees' attitudes and behaviors, such as organizational identification and job performance. Most previous studies on socially responsible HRM focused on its relationship with employee organizational citizenship behavior, organizational commitment, job satisfaction, well-being, and turnover intention. For example, Shen and Zhu (2011) demonstrated that socially responsible HRM is positively related to organizational commitment. Sobhani et al. (2021) showed that socially responsible HRM has a significant positive impact on employee organizational citizenship behavior and a significant negative influence on turnover intention. Luu et al. (2022) argued that socially responsible HRM practices are positively associated with job crafting based on the mutual gains perspective in public management. In addition, there has been a recent research trend of socially responsible HRM due to its positive impacts on both individual employees and organizations (Del-Castillo-Feito et al., 2022; Omidi and Dal Zotto, 2022). However, very little is known about the direct effect of employees' perception of their organization's socially responsible HRM on their job performance and the indirect influence via their organizational identification. Therefore, this study seeks to fill these research gaps by examining the impact of perceived socially responsible HRM on job performance and the mediating role of organizational identification in the relationship between these two variables.

Furthermore, in the context of a global crisis, employees are more fearful of external threats and require a greater level of support and assistance from their organization (Cheng and Kao, 2022; He et al., 2021; Sorribes et al., 2021). The COVID-19 pandemic has been the largest worldwide catastrophe since World War II. This crisis has significantly impacted human health and wreaked havoc on economies, labor sectors, and individual employees (International Labor Organization, 2020). During a crisis, a company's proactive reactions allow employees to return to work and focus on the organization's goals, as well as reassure them that they can trust the firm (Watkins et al., 2015). Markovits et al. (2014) pointed out that employees' self-regulation processes are affected by an economic crisis, and these mechanisms explain their attitudinal changes. However, previous studies claimed that research on the dynamic interactions between contextual factors, individual intentions, behavior, and decisions was restricted and lacked coherent explanations and prediction hypotheses (Myer and Moore, 2006; Wu et al., 2012). Given that system study regards people as part of the system, most crisis studies have concentrated on organizational and management levels, not individual layers (Wu et al., 2012). Nonetheless, employees' willingness and responses are, nonetheless, greatly impacted by the features of their workplace. Therefore, it is critical to study individual employee's organizational identification and job performance during a global crisis to fill the research gap regarding how a global crisis affects people in their workplace.

Employees' perceived organizational response to a crisis refers to employees' general perception that their organization has reacted appropriately and effectively in assisting and supporting them during the crisis (Mao et al., 2020; Vo-Thanh et al., 2020; Wiitala and Mistry, 2022). Employees' view of organizational support, or the extent to which the company values members' efforts and concerns about their well-being, is regarded as a result of the organization's supportive activities during a crisis (Watkins et al., 2015). Therefore, during a global crisis such as the COVID-19 pandemic, organizations could combine socially responsible HRM practices and employees' identification with organizational response to a crisis to promote employees' positive psychology, attitudes, behavior, and job performance, consequently ensuring the organization's sustainable development in the long term. Especially during a crisis, employees' organizational identification and job performance is the key to helping organizations overcome difficult situations (Lian et al., 2022; Vo-Thanh et al., 2020). Moreover, the findings of Lian et al. (2022) indicated that the COVID-19 pandemic might not degrade organizational identity, but instead, boost it. As employers respond to external risks with layoffs or pay cutbacks, workers' organizational identity might be at risk. In contrast, COVID-19 pandemic-related uncertainty reveals commonalities between workers and as well as fosters a greater sense of “organizational we-ness”, hence enhancing their organizational identification. To the best of our knowledge, there have been no other studies that evaluate the effect of the interaction between employees' perceived organizational response to a crisis and organizational identification on job performance during a global crisis such as the COVID-19 pandemic. Therefore, this is one of the first empirical studies that evaluates this subject.

## 2 Theoretical background and hypotheses development

### 2.1 Perceived socially responsible human resource management and job performance

Socially responsible HRM is a set of HRM practices that not only comply with labor standards set by the International Labor Organization but also exceed regulations required to address the workers' interests, needs, and benefits, as well as those of other stakeholder groups and the wider community (Shen and Zhu, 2011). In other words, socially responsible HRM practices comprise three components: legal compliance HRM, employee-oriented HRM, and general CSR facilitation HRM (Luu, 2021; Shen and Benson, 2016; Shen and Zhu, 2011). According to studies in the HRM literature, supportive HRM policies can be considered as a sign that the corporation is concerned about its employees, and consequently leads to employees' positive outcomes, such as organizational citizenship behaviors, work engagement, job performance, and employee loyalty (De la Rosa-Navarro et al., 2020; Kloutsiniotis and Mihail, 2020). For instance, Shen and Zhu (2011) demonstrated that socially responsible HRM positively relates to organizational commitment. Furthermore, Kundu and Gahlawat (2015) indicated that socially responsible HRM has a significant positive influence on employees' job satisfaction and a significant negative influence on employees' intention to quit. Additionally, Jia et al. (2019) demonstrated that socially responsible HRM enhances frontline employees’ respect for and trust in their organization and further encourages their knowledge sharing.

Employee job performance is *among the most important concerns* for a manager, *as it can increase an organization's overall performance, both* directly and indirectly (Chiang and Hsieh, 2012; Khtatbeh et al., 2020; Vu et al., 2022). Job performance is defined as *the “scalable actions, behaviors, and outcomes that employees engage in or bring about that are linked with and contribute to organizational goals”* (Viswesvaran and Ones, 2000, p. 216). Studies on the antecedents of job performance illustrated that various organizational factors lead to employee job performance (Pandey, 2019; Rich et al., 2010). Several of these factors, such as perceived organizational support, organizational resource, and manager feedback, are related to socially responsible HRM. Shao et al. (2019) suggested that perceived socially responsible HRM affects task performance and social performance through both cognitive and affective paths. Furthermore, Manzoor et al. (2019) demonstrated that sustainable HRM practices, such as selection, participation, and employee empowerment, have a significantly positive impact on employee job performance. Additionally, Shen and Benson (2016) showed that organization-level socially responsible HRM is an indirect determinant of employees' task performance.

According to the norm of reciprocity in the social exchange theory (Blau, 1964), socially responsible HRM practices may foster positive attitudes and behaviors among workers (Aryee et al., 2002; Luu, 2021). This theory emphasizes the mutually dependent transactions in which one party offers a resource, then the other party reciprocates the favor from a sense of obligation, resulting in a new trade cycle (Karim et al., 2021). Social exchange is grounded on a dispersed responsibility to reciprocate and is based on a long-term exchange of favors that prevents accounting (Aryee et al., 2002). A positive reciprocity intention entails the tendency to return good treatment for favorable treatment. This theory has been used to explain the effect of socially responsible HRM on employees' positive attitudes and behaviors (Jia et al., 2019; Luu, 2021; Newman et al., 2016). Indeed, socially responsible HRM practices focus on ensuring employees' rights and well-being, catering to their demands, providing opportunities for learning and growth, and encouraging employees to speak up via workplace democracy and power-sharing. In addition, socially responsible HRM encourages employees to participate in CSR activities and rewards their contribution to the CSR initiatives of their organization (Barrena-Martinez et al., 2019; Shen, 2011; Zhao et al., 2022). Such positive organizational actions may persuade employees to view themselves as valuable members. This leads to an underlying need to respond by engaging in activities and behaviors that benefit the organization, such as job performance (Luu, 2021; Luu et al., 2022; Newman et al., 2016), according to the reciprocity principle of the social exchange theory. Consequently, it can be argued that when employees perceive their organization's HRM to be socially responsible, it may promote their job performance. Therefore, hypothesis 1 is:

H1 Perceived socially responsible HRM positively influences employee job performance.

### 2.2 Perceived socially responsible HRM and organizational identification

Many definitions of organizational identification have been proposed. For example, organizational identification is defined as “a specific form of social identification” and “the perception of oneness with, or belongingness to an organization, where the individual defines him or herself in terms of the organization(s) in which he or she is a member” (Ashforth and Mael, 1989, p. 22), as well as the extent to which a person defines himself or herself by “the same attributes that he or she believes define the organization” (Dutton et al., 1994, p. 240). Regardless of their differences, all these definitions indicate that employees have related their membership to their self-concept, cognitively (e.g., considering themselves a part of the corporation; integrating organizational ideals), emotionally (prestige in belonging), or both. In other words, organizational identification alludes to the psychological bond that exists between an employee and an organization.

Employees’ perceived socially responsible HRM of their organization may have a positive impact on their organizational identification based on the principle of the social identity theory. Indeed, socially responsible HRM practices can help develop organizational reputation and prestige (Barrena-Martinez et al., 2019; Del Mar Ramos-González et al., 2021; Sobhani et al., 2021). This organizational reputation and prestige can improve employee organizational identification since, according to the social identity theory (Ashforth and Mael, 1989; Tajfel et al., 1979), people will identify more strongly with their organization when they believe it has a positive reputation. Bauman and Skitka (2012) and Newman et al. (2016) argued that this belief could make an employee feel proud to work for the organization, enhance their self-concept, and improve their identification with the organization.

In addition, considering that identification is a cognitive notion that reflects the extent to which the organization is absorbed into self-conceptualization, employees' organizational identification is viewed as a dependent factor, such as perceived commonality and a shared morality with the corporation (Wang et al., 2017). Smidts et al. (2001) demonstrated that employees' self-concepts are positively influenced by organizations with great prestige, and their identification with the organization is enhanced. According to Shen and Benson (2016), people are willing to appraise their organization based on its CSR actions, as CSR has primarily established a global social norm. Employees will respond positively to an organization's effective participation in CSR, resulting in stronger identification with the enterprise (Berger et al., 2006; Upham, 2006; Wang et al., 2017). Therefore, following the social identity theory (Ashforth and Mael, 1989; Tajfel et al., 1979), it is argued that when employees perceive that their organization employs socially responsible HRM practices with good values and moral ethics, they would feel more identified with their organization.

The positive influence of socially responsible HRM practices on employees' organizational identification could also be explained by the social exchange theory (Blau, 1964). As mentioned above, according to this theory, in a relationship, two parties need to obey the rule of reciprocity. Socially responsible HRM practices prioritize the respect of employees' rights, the satisfaction of their needs, the offering of learning and development opportunities, the promotion of workplace democracy, and the recognition of employees' contributions (Barrena-Martinez et al., 2019; Shen, 2011). Such positive organizational initiatives may enable employees to believe they are valuable members of their organization, thereby enhancing their sense of self-worth. Consequently, by following the reciprocity principle of the social exchange theory, employees may respond by increasing their identification with the organization (Fuller et al., 2006). Moreover, organizations’ socially responsible HRM practices may generate a healthy and resourceful workplace setting, which can make the organization become a favorable place to work and develop employee organizational identification (Chughtai, 2016; Schaubroeck et al., 2011). Therefore, employees will build higher degrees of organizational identification when they reciprocate by engaging psychologically in an organization which has CSR SR-HRM practices that benefit them directly (He et al., 2014; Newman et al., 2016). Hence, hypothesis 2 is:

H2 Perceived socially responsible HRM positively influences organizational identification.

### 2.3 The mediating role of organizational identification

Drawing on the social identity theory, the current study proposes that organizational identification will lead to increased job performance. Organizational identification, which refers to the “perception of oneness with or belongingness to the organization” (Ashforth and Mael, 1989, p. 22), may encourage employees to adopt their organization's perspective and to regard their organization's values and goals as their own (Chughtai and Buckley, 2010; Dutton et al., 1994). Previous research has shown that employees with a high level of organizational identification are more likely to exert significant effort, contribute their greatest to work, collaborate, engage in extra-role behaviors, feel more satisfied with their job, have lower turnover intentions and actual turnover, and are predicted to perform work well since they are filled with a deep sense of belonging (e.g., Chughtai and Buckley, 2010; Edwards and Peccei, 2010; Mael and Ashforth, 1995).

According to the social identity theory, organizational identification has an important strategic significance since the more that employees identify with the organization, the more they are willing to be involved in and devote their efforts to their organization to achieve its objectives (Callea et al., 2019). Moreover, Astakhova and Porter (2015) explained that according to self-determination theory, integration occurs when “identified regulations are fully assimilated to the self, which means they have been evaluated and brought into congruence with one's other [organizational] values and needs” (Ryan and Deci, 2000, p. 73), and the relationship between organizational identification and job performance may resemble identified regulations. These employees will work cooperatively and exert a significant amount of effort. Moreover, according to Vroom's (1964) expectancy theory, employees who have a higher level of identification with the organization are more likely to have more instrumentalities in terms of effort and more positive valence values in performing good job tasks. Therefore, hypothesis 3 is:

H3 Organizational identification positively influences job performance.

It was previously established that perceived socially responsible HRM and organizational identification are two crucial variables that contribute to an increase in employees' job performance. In the interaction between these three variables, organizational identification is anticipated to serve as a mediator between socially responsible HRM and job performance. Indeed, it can be argued that perceived socially responsible HRM may enhance employees' organizational identification, which, in turn, enhances their job performance. In addition, perceived socially responsible HRM can enhance employee job performance. Consequently, organizational identification can mediate the link between perceived socially responsible HRM and employees' job performance. The relationships among perceived socially responsible HRM, organizational identification, and employees' voice behavior can also be explained by the social identity theory. Socially responsible HRM practices can build organizational norms and values (Newman et al., 2016; Sobhani et al., 2021). These norms and values are integrated with employees' organizational identification into their self-concept. Given that they have assimilated these standards into their self-concept, workers who deeply identify with their organization will think and act in accordance with their organization's norms and values (Callea et al., 2019). Previous studies also demonstrated that organizational identification mediates the relationship between variables and job performance. For example, Walumbwa et al. (2011) demonstrated that organizational identification plays the role of a full mediator in the relationship between ethical leadership and employee performance. Wang et al. (2017) showed that when there is a strong CSR awareness, workers would develop greater organizational identification and in turn demonstrate positive outcomes such as in-role job performance and helping behaviors. In other words, if CSR is seen favorably, employees will experience a sense of engagement with the company, resulting in greater organizational identification and eventually improved performance. Therefore, hypothesis 4 is:

H4 Organizational identification plays a mediating role in the nexus between employees' perceived socially responsible HRM and job performance.

### 2.4 The moderating effect of employees’ perceived organizational response to a global crisis

Every crisis generates a large amount of uncertainty for stakeholders of an organization, including employees (Markovits et al., 2014; Ulmer et al., 2017; Wiitala and Mistry, 2022). Employees are tested when a crisis happens since their jobs could be crucial or even harmful. When an organization is confronted with a frightening circumstance of crisis, management begins to ask: “Are the employees more or less willing to contribute? What factors affect the employees' willingness to accept assigned jobs?” (Wu et al., 2012, p. 2698). During such a crisis, a company's proactive reactions allow workers to continue working and focus on the institution's goals, as well as reassure workers that they can trust the firm (Watkins et al., 2015). Therefore, the company should adopt immediate and appropriate measures to rebuild employee confidence and reassure them (Vo-Thanh et al., 2020). Furthermore, Hurley-Hanson (2006) and Mao et al. (2020) stated that organizations need to have a comprehensive scheme to respond to all types of unexpected disasters or crises.

Previous research has shown that employees' perceived organizational support can act as a buffer against employee mental health and job dissatisfaction as a result of a crisis or tragedy (Kim and Niederdeppe, 2013; Sanchez et al., 1995; Vo-Thanh et al., 2022). In contrast, when employees become aware of the lack of attention by their employers to their job, their psychology, and their situation during the crisis, they could feel angry. For example, during the event of the September 11^th^, 2001, attack in America, many workers felt betrayed by their companies and questioned why more was not done to ensure their individual security. Several individuals hoped that their employers would alleviate their anxieties and help them feel protected; however, many found employers' responses to be just “cold and impersonal” (Mainiero and Gibson, 2003, p. 133). These remarks reveal a mix of anger and vulnerability, as well as a severe loss of trust among employees in their employers and organizations. They express a desire to be linked with others, but they believe the organization will be unable to fulfill this need. These adverse perceptions and psychologies could cause employees’ negative attitudes and behaviors in the workplace.

According to Schouten et al. (2004), organizational crisis preparation is crucial for a variety of reasons from a business standpoint. Mitigation of the physical, psychological, and business effects of crises; legal duties to engage in disaster preparation; and the positive influence of such activities on workers’ relationships with their workplace are all worth mentioning. Firms that engage in these activities and suffer a crisis can anticipate increases in work satisfaction, staff retention, productivity, and health problems, as well as a reduction in potential legal exposure. Bardoel et al. (2014) believe that resilience-enhancing HRM strategies will have a direct positive relationship with good employee outcomes such as organizational commitment, work satisfaction, and work performance, in addition to the resilience dimension of psychological capital.

Employees' perceived organizational response to a crisis reflects their overall impression of their corporation's suitable and effective response to the situation (Mao et al., 2020; Watkins et al., 2015). The more satisfied workers are with the company's reactions to a crisis, the more they believe the firm, feel safe and protected in their employment, and are enthusiastic and inspired to make a significant contribution to the work, all of which result in greater productivity. Furthermore, if employees are satisfied with their firm's concentration and dedication to overcoming a crisis such as the COVID-19 pandemic, they should realize change and be persuaded to embrace the endeavor and nurture the achievement of work duties (Vo-Thanh et al., 2020). This conforms to the organizational support theory's guiding principle, which is grounded in the social exchange theory that views employment as a transaction of employees' effort and allegiance to the organization's real benefits and social resources (Cropanzano and Mitchell, 2005).

Previous studies also indicated that employees' perceived organizational response to a crisis or employees' perception of organizational support during a crisis served as a moderating factor in the research field of HRM. For example, Vo-Thanh et al. (2020) demonstrated that employees' satisfaction with the organization's responses to the COVID-19 pandemic moderates the positive connection between employees' awareness of the health risk of the COVID-19 pandemic and their job insecurity. Their study also revealed that staff members' satisfaction with the firm's responses to COVID-19 acts as a moderating factor in the negative association between perceived job insecurity and job performance, such that this association is poorer as workers' satisfaction with the firm's COVID-19 reactions increases. Nikandrou and Tsachouridi (2015) found that the perception of organizational virtuousness moderates the impacts of a financial crisis on employee job satisfaction and intention to leave.

According to the principle of the organizational support theory based on the social exchange theory (Cropanzano and Mitchell, 2005), employees' perceived organizational response to a crisis could interact with their organizational identification to influence their job performance. This theory proposes that employees will return their organizations' good deeds with favorable job attitudes and actions if they believe their organization values and treats them fairly (Aryee et al., 2002). When the organization adopts an appropriate response to a crisis, workers will believe that their employer cares about their well-being, employment, and health during a crisis. This, in turn, increases the likelihood that employees will feel safe at work and demonstrate positive attitudes and behaviors, such as organizational citizenship behaviors, organizational commitment, job satisfaction, work engagement, and work performance (Vo-Thanh et al., 2020; Watkins et al., 2015). Moreover, according to the conservation of resource theory (Hobfoll, 1989), workers' psychophysiological pressure due to a depletion of resources produced by a crisis can be mitigated if they are happy with their firm's reactions to a crisis such as the COVID-19 pandemic. This study proposed that during the challenging period of the COVID-19 crisis, employees' perceived organizational response to a crisis would help mitigate the impact of their organizational identification on their job performance. Therefore, hypothesis 5 and hypothesis 6 are:

H5 Perceived organizational response to a crisis positively influences job performance.

H6 Perceived organizational response to a crisis moderates the positive association between organizational identification and job performance.

**Figure 1:**
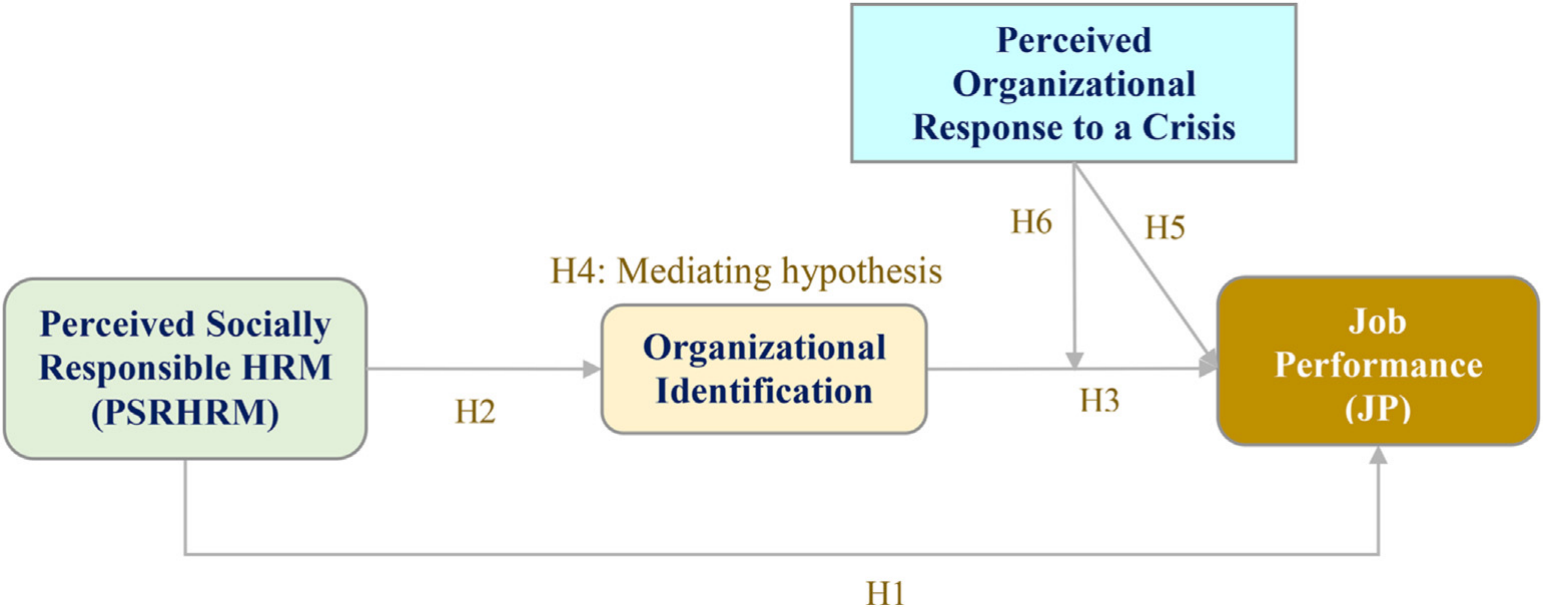
Research model.

The research model is shown in Figure 1.

## 3 Research methodology

### 3.1 Sampling and data collection

This survey collected data from Vietnamese full-time employees (the top management level was excluded) working in the following industries in Vietnam: trade, wholesale, and retail; transportation, warehousing, and logistics; construction and real estate; tourism, restaurant, leisure, and hotel services; and manufacturing or processing (e.g., textile, footwear, electronic, food processing). According to the International Labour Organization (2020) and the World Bank (2021), the above industries have been more heavily impacted by COVID-19 than others. This study used a filter question – *“Are you currently working in one of the following industries…?”* to ensure that we selected suitable employees as the subjects of this research. Concerning the adaption of the scales, following the suggestions of Hardesty and Bearden (2004), this assessment required the participation of five HRM specialists. The invited experts reviewed the constructs’ items, adjusted them, and made recommendations to ensure content and face validity. To guarantee linguistic equality, the survey instrument was prepared using the back-translation process (Brislin, 1970): after being translated into Vietnamese from English, the measuring scales were subsequently translated back into English.

An online questionnaire was created in Google Forms and sent via email and LinkedIn to the prospective respondents. The invitation included details about the study's goals, survey instructions, and confidentiality pledges. This method of data gathering was chosen to reduce the risk of infection for both investigators and participants. All the respondents answered the questionnaires voluntarily. An online informed consent was obtained from all participants prior to proceeding with the survey.

In this study, the main data were collected from an online survey conducted between November 2021 and March 2022 in Vietnam. During this period, Vietnam experienced the fifth *wave* of the *COVID-19* pandemic in a complex and unprecedented situation. To reduce common method bias (CMB) associated with one-time data collection (Podsakoff et al., 2003), this study conducted a two-stage survey. In the initial stage, participants were asked to supply demographic and work-related information, as well as information on perceived socially responsible HRM, and perceived organizational response to the COVID-19 pandemic. A total of 1,000 questionnaires were sent to the prospective respondents in the first-stage survey, and a total of 507 completed responses were obtained. In the second stage, which took place four weeks later, data on organizational identification and job performance were collected. The data from the two stages were matched by assigning each respondent a code. The final sample consisted of 367 full-time Vietnamese employees.

### 3.2 Measurement scales

To measure the model's variables, both dependent and independent, 5-point Likert scales ranging from “strongly disagree” (1) to “strongly agree” (5) were used. There were four constructs in the research framework (Figure 1) (perceived socially responsible HRM, organizational identification, perceived organizational response to a crisis, and job performance), which were evaluated using questions developed from previous research and adapted to fit the context of the present study.

The scale of perceived socially responsible HRM was adapted from Shen, 2011, Shen and Zhu, 2011 and Luu (2021). It is a multidimensional, second-order formative construct assessed via three sub-constructs: perceived legal compliance HRM practices (five items); perceived employee-oriented HRM (five items); and perceived general CSR facilitation HRM (three items). In addition, to validate the formative higher-order measurement of socially responsible HRM by employing a redundancy analysis (Sarstedt et al., 2019), this study developed a global question for this construct. Organizational identification was measured using five items adapted from Mael and Ashforth (1995). Perceived organizational response to a crisis was assessed using three measures derived from Watkins et al. (2015) and Vo-Thanh et al. (2020) in the context of the COVID-19 crisis. Job performance was measured using six items adapted from Chiang and Hsieh (2012) and Vu et al. (2022).

## 4 Analysis and findings

### 4.1 Descriptive analysis

Table 1 illustrates the characteristics of respondents, namely, age, gender, position, industry, type of work contract, and work organization size. The majority of respondents were aged 30 and under (54.8%), followed by those aged from 31 to 40 (32.4%). Females accounted for 60.5%, males accounted for 38.7%, and others accounted for 0.8%. Respondents were from different industry sectors: 37.6% worked in trade, wholesale, and retail; 21% worked in manufacturing or processing (textile, footwear, electronic, food processing); 15.5% worked in tourism, restaurant, leisure, and hotel services; 12.5% worked in transportation, warehousing, and logistics; and 13.4% worked in construction and real estate. The majority of respondents had an indefinite contract (40.1%), followed by those who had a contract for over 2 years (32.1%). Respondents worked in companies of different sizes, for example, 28.6% worked in companies with less than 50 employees, and 11.4% worked in companies with 501–1,000 employees.

**Table 1:** Descriptive information of sample (*n* = 367).

Category	n	%	Category	n	%
*Age*			*Work organization size (employees)*		
≤30	201	54.8	<50	105	28.6
31–40	119	32.4	51–100	55	15.0
≥41	47	12.8	101–200	47	12.8
*Gender*			201–500	48	13.1
Male	142	38.7	501-1,000	42	11.4
Female	222	60.5	1,001–2,000	25	6.8
Others	3	0.8	>2,000	45	12.3
*Position*			*Industry*		
Non-managerial employee	277	75.5	Trade, wholesale, and retail	138	37.6
First-line manager	48	13.1	Transportation, warehousing, and logistics	46	12.5
Middle manager	42	11.4	Construction and real estate	49	13.4
*Type of work contract*			Tourism, restaurant, leisure, and hotel services	57	15.5
A contract for under 1 year	11	3.0	Manufacturing or processing (Textile, footwear, electronic, food processing, etc.)	77	21.0
A contract for 1–2 years	91	24.8			
A contract for over 2 years	118	32.1			
An indefinite contract	147	40.1			

### 4.2 Assessment of the measurement model

First, this study tested the reliability and validity of the measurement model. As shown in Table 2, perceived socially responsible HRM was formative; therefore, a reliability analysis utilizing rho_A, composite reliability (CR), and average variance extracted (AVE) was not applicable. Regarding the reflective sub-constructs of perceived socially responsible HRM (perceived legal compliance HRM, perceived employee-oriented HRM, perceived general CSR facilitation HRM) and the three remaining reflective constructs (organizational identification, perceived organizational response to a crisis, and job performance), all these observed variables had outer loadings between 0.63 and 0.92, which were over the 0.50 threshold (Hulland, 1999). In addition, their relevant bootstrapped *t*-values exceeded 1.96 and fell within the statistically significant range of 8.61–68.17. The value of AVE of the reflective variables ranged from 0.57 to 0.78, all exceeding the 0.50 threshold, indicating adequate convergent validity. The rho_A values of constructs were higher than the threshold of 0.7 (Dijkstra and Henseler, 2015). Additionally, the CR indices of the reflective variables were between 0.83 and 0.94, all exceeding the 0.70 threshold, demonstrating the reliability of the measured data (Hair et al., 2019).

**Table 2:** Constructs’ measurement.

Measurement items	Loadings	*t*-value
**Perceived Socially Responsible HRM**^*f*^		
***Perceived Legal Compliance HRM (Cronbach's alpha = 0.85; rho_A = 0.86; CR = 0.89; AVE = 0.57)***		
• “My organization ensures equal opportunity for employees in HRM”	0.786	32.386
• “Employees in my organization are paid above minimum wages and based on their performance”	0.802	30.909
• “My organization complies with the regulations regarding the contract labor, working hours, and compulsory social benefits”	0.747	26.281
• “My organization does not employ child labor or forced labor”	0.633	8.609
• “My organization has clear and detailed regulations on occupational health and safety”	0.756	23.761
• “My organization appoints staff monitoring labor standards in business partners; for example, suppliers and contractors”	0.796	29.221
***Perceived Employee-oriented HRM (Cronbach's alpha = 0.84; rho_A = 0.85; CR = 0.89; AVE = 0.61)***		
• “My organization adopts flexible working hours and employment programs achieving work-life balance”	0.688	17.994
• “My organization provides adequate training and development opportunities to employees”	0.789	36.657
• “Bottom-up voice is stimulated in the organization”	0.849	53.694
• “Employees are allowed to participate in decision making and total quality management; and their suggestions and ideas are appreciated by managers”	0.778	27.509
• “Unions can represent and protect workers' rights and can be involved in determining labor terms”	0.804	34.453
***Perceived General CSR Facilitation HRM (Cronbach's alpha = 0.82; rho_A = 0.83; CR = 0.89; AVE = 0.74)***		
• “My organization appoints adequate staff implementing general CSR initiatives (toward shareholders, community, environment, employees, customer, and other partners)”	0.846	45.249
• “My organization enforces employee participating on CSR activities; rewards employees who contribute to environmental protection, charity, communities, and other CSR activities”	0.891	72.131
• “My organization gives equal opportunity employment to all candidates, including those who are in difficulty and who are local”	0.835	41.458
**Organizational Identification (Cronbach's alpha = 0.85; rho_A = 0.87; CR = 0.89; AVE = 0.62)**		
• “When someone criticizes the organization, it feels like a personal insult”	0.710	16.387
• “I am very interested in what others think about the organization”	0.694	15.475
• “When I talk about the organization, I usually say ‘we’ rather than ‘they’”	0.806	27.572
• “The organization's successes are my successes”	0.860	51.634
• “When someone praises the organization, it feels like a personal compliment”	0.853	46.581
**Perceived Organizational Response to a Crisis (Cronbach's alpha = 0.89; rho_A = 0.90; CR = 0.93; AVE = 0.82)**		
• “I am satisfied with the way that my employer responded to crisis by prompt and appropriate plans and scenarios”	0.909	53.951
• “I am satisfied that my organization's management board did everything that it could have in response to crisis”	0.931	115.81
• “I am satisfied with the way that my organization's management board took care of its employees' needs and difficulties resulting from crisis”	0.882	40.292
**Job Performance (Cronbach's alpha = 0.92; rho_A = 0.93; CR = 0.94; AVE = 0.73)**		
• “I fulfill my job responsibilities”	0.808	25.663
• “I meet the performance standards and expectations of the job”	0.863	46.634
• “My performance level satisfies my manager”	0.886	63.250
• “I perform better than many other ones who perform the same job”	0.787	26.874
• “I have adequate competencies to carry out my work effectively”	0.879	59.029
• “I produce high-quality work”	0.889	68.174
• “I fulfill my job responsibilities”	0.808	25.663

Notes*:*^f^: CR, rho_A, and AVE are not appropriate for the formative construct.

To assess discriminant validity, this study followed the approach proposed by Fornell and Larcker (1981). Except for the formative construct (perceived socially responsible HRM), Table 3 revealed that the square root of AVE of each reflective construct is greater than its highest correlation with other constructs (Fornell and Larcker, 1981). Additionally, the HTMT values presented in Table 3 varied from 0.51 to 0.77, substantially below 0.85, demonstrating stronger support for discriminant validity (Henseler et al., 2015).

**Table 3:** Correlations between constructs, Fornell-Larcker criterion, and HTMT values.

Variable	*LC*	*EO*	*GC*	PSRHRM^s^	OI	JP	PSOR
*LC*	**0.76**	*0.66*	0.57	*N/A*	*0.54*	*0.51*	*0.69*
*EO*	0.57^c^	**0.78**	*0.75*	*N/A*	*0.56*	*0.48*	*0.65*
*GC*	0.49^c^	0.63^c^	**0.86**	*N/A*	*0.53*	*0.51*	*0.67*
PSRHRM^s^	0.61^c^	0.88^c^	0.78^c^	**N/A**	*0.62*	*0.58*	*0.77*
OI	0.61^c^	0.48^c^	0.44^c^	0.55^c^	**0.79**	*0.56*	*0.53*
JP	0.46^c^	0.42^c^	0.46^c^	0.53^c^	0.51^c^	**0.85**	*0.55*
PSOR	0.61^c^	0.57^c^	0.58^c^	0.70^c^	0.55^c^	0.51^c^	**0.88**

Notes: LC: Perceived Legal Compliance HRM; EO: Perceived Employee-oriented HRM; GC: Perceived General CSR Facilitation HRM; PSRHRM: Perceived Socially Responsible HRM; OI: Organizational Identification; PSOR: Perceived Organizational Response to a Crisis; JP: Job Performance.

LC, EO, and GC are first-order reflective components of SRHRM; Bold diagonal is the square root of AVE; ^s^: second-order formative construct; values below the diagonal: the correlations among constructs; values above the diagonal (italic): HTMT ratio; ^c^: Correlation is significant at the 1% level (2-tailed t-test); N/A: HTMT values are not applicable for the relationship between first-order reflective constructs and second-order formative constructs.

### 4.3 Common method bias analysis

Given that this study employed cross-sectional data, Harman's single-factor evaluation developed by Podsakoff et al. (2003) was utilized to test for the problem of CMB. The objective was to determine whether a single variable accounted for more than 50% of the variance, which could be problematic since it implies the presence of CMB. The issue of CMB was eliminated after it was determined that the first element accounted for just 37.26% of the variance (<50%). In addition, as mentioned above, the square root of AVE of each construct was greater than its highest correlation with other constructs (see Table 3). Thus, discriminating validity was assured. In addition, the independent variables' variance inflation factor (VIF) values should be considered since they can identify potential multicollinearity issues (O'Brien, 2007). In this study, all the inner VIF values, which ranged from 1.07 to 2.33, were close to the ideal threshold value of 3.00 (Hair et al., 2019), indicating that multicollinearity was not a major issue.

### 4.4 Evaluating the measurement structural model and results of hypothesis testing

Partial least squares (PLS) analysis with SmartPLS software (version 3.2.7) was used in this research to verify the framework and the proposed hypotheses.

Hair et al. (2019) suggested that the coefficients of determination $R^{2}$ that reflected the amount of explained variance of each endogenous latent variable were the most important criterion for evaluating the PLS model. According to Chin (1998), $R^{2}$ above the cut-offs of 0.67, 0.33, and 0.19 are described as “substantial,” “moderate,” and “weak,” respectively. In this study, the R^2^ values for organizational identification and job performance were 0.31 and 0.41, respectively. Therefore, these values were considered high and acceptable. For a specific endogenous construct, $Q^{2}$ values were greater than zero, showing the structural model's prediction accuracy for constructs. $Q^{2}$ values were greater than 0, 0.25, and 0.50, respectively, indicating small, medium, and substantial predictive meaning of the PLS-path model (Hair et al., 2019). The results of this study showed that the $Q^{2}$ values of organizational identification and job performance were 0.18 and 0.29, respectively. Therefore, these values are acceptable.

The parameter estimates of the path between research constructs were used to test the structural model and its research hypotheses. The statistical significance of each path coefficient for hypothesis testing was determined by using a nonparametric bootstrapping process with 5,000 sub-samples using a sample of 367.

H1 proposes that employees’ perceived socially responsible HRM positively influences their job performance. The *ß* coefficient for the path of perceived socially responsible HRM–job performance was 0.22 and statistically significant at the 1% level (*t*-value = 3.49), supporting this hypothesis. H2, which posits that perceived socially responsible HRM positively influences organizational identification, was supported, as the *ß* coefficient for the perceived socially responsible HRM–organizational identification path was 0.55 and significant at the 1% level (*t*-value = 11.81). H3 proposes that organizational identification positively impacts job performance. This hypothesis was confirmed, as the organizational identification–job performance path was significant at the 1% level (*ß* = 0.24, *t*-value = 4.41).

H4 suggests that organizational identification acts as a mediator in the correlation between perceived socially responsible HRM and job performance. Table 4 showed that the indirect relationship between perceived socially responsible HRM and job performance was statistically significant at the 1% level (*ß* = 0.31, *t*-value = 4.41, CI = [0.07; 0.19]) but higher than the direct correlation between two above constructs (*ß* = 0.22, *t*-value = 3.49). This result indicated that organizational identification plays a mediating role in the nexus between perceived socially responsible HRM and job performance, supporting hypothesis H4. The Sobel test was then used in this study to confirm this result. A further confirmation of H4 regarding the mediating effect of organizational identification in the link between perceived socially responsible HRM and job performance was provided by the Sobel test statistic of 4.15 and being statistically significant at the 1% level (two-tailed t-test).

**Table 4:** The results of hypothesis testing.

H	Relations	Path coefficient	t-value	p-value
H1	PSRHRM → JP	0.22	3.49	0.000
H2	PSRHRM → OI	0.55	11.81	0.001
H3	OI → JP	0.24	4.41	0.000
H4	PSRHRM → OI → JP	0.31	4.31	0.000
H5	PSOR → JP	0.18	2.84	0.004
H6	PSOR∗OI → JP	-0.14	3.87	0.000

Note: PSRHRM: Perceived Socially Responsible HRM; OI: Organizational Identification; PSOR: Perceived Organizational response to a crisis; JP: Job Performance.

H5 conjectures that employees’ perceived organizational response to a crisis positively influences their job performance. The *ß* coefficient for the path of perceived organizational response to a crisis–job performance was 0.18 and statistically significant at the 1% level (*t*-value = 2.84); thus, H5 was supported.

H6 suggests that perceived organizational response to a crisis moderates the nexus between organizational identification and job performance. To test this moderating hypothesis, this study created an interaction term, PSOR∗OI, after mean centering the moderator (i.e., perceived organizational response to a crisis) and the independent variable (i.e., organizational identification) to avoid multicollinearity (Aiken et al., 1991). Remarkably, this study demonstrated that perceived organizational response to a crisis negatively moderates the impact of organizational identification on job performance (β = −0.14, *t*-value = 3.87); thus, H6 was supported.

**Figure 2:**
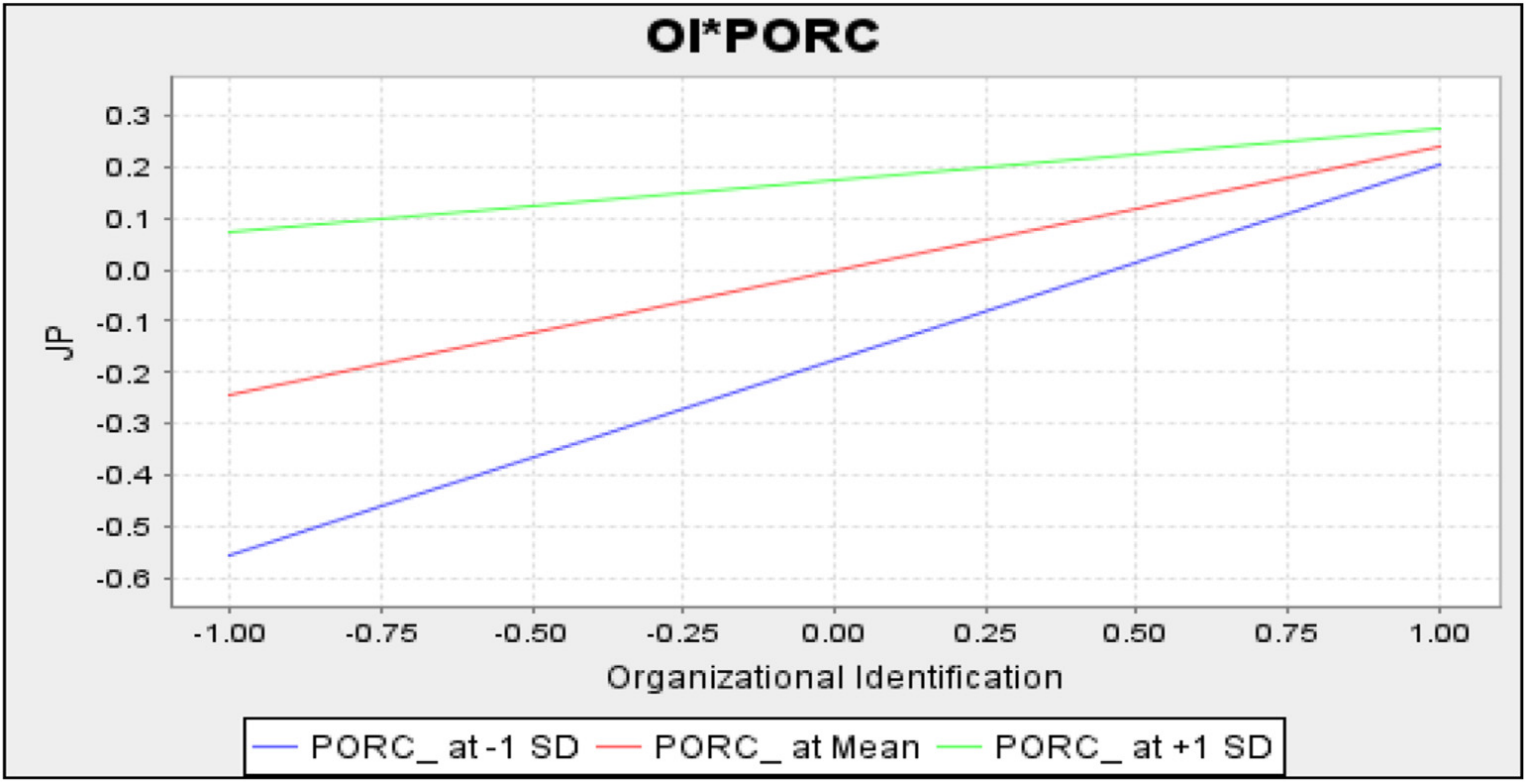
The moderating effect of perceived organizational response to a crisis in the relationship between organizational identification and job performance.

To better understand the interaction effect, a simple slope test was conducted (Aiken et al., 1991). The interaction plot in Figure 2 showed that the slopes of the regressions are less positive under the high level of the moderator variable (i.e., perceived organizational response to a crisis) than under the low level of the moderator variable. Therefore, organizational identification has a weaker effect on the job performance of employees who perceive high organizational responses to a global crisis than those who perceive low organizational responses to a global crisis. In other words, the effect of organizational identification on job performance is stronger at a low level than at a high level of perceived organizational response to a crisis.

To further confirm the moderating role of perceived organizational response to a crisis, this study carried out comparisons between groups by employing the permutations and **multi-group analysis (**MGA) procedure. Before running these tests, the data of perceived organizational response to a crisis was divided into two groups, i.e., high perceived organizational response to a crisis and low perceived organizational response to a crisis.

Prior to running the MGA, the measurement invariance of composite models (MICOM) must be evaluated (Henseler et al., 2016). The MICOM consists of three phases: “(1) configural invariance, (2) compositional invariance, and (3) the equality of composite mean values and variances” (Henseler et al., 2016, p. 406). Partial invariance can be established by observing composite invariance, as demonstrated in Table 5 of the MICOM process findings. Partial invariance is adequate for utilizing the permutations and MGA method to evaluate differences across groups (Cachón-Rodríguez et al., 2021; Hair et al., 2018; Rasoolimanesh et al., 2017).

**Table 5:** Results of invariance measurement testing using permutation.

Construct	Configural invariance (Same algorithms for both groups)	Compositional invariance (Correlation = 1)		Partial measurement invariance established	Equal mean value		Equal variance		Full measurement invariance established
		C = 1	Confidence Interval (CIs)		Differences	Confidence Interval (CIs)	Differences	Confidence Interval (CIs)	
OI	Yes	0.999	[0.994, 1.000]	Yes	-1.026	[-0.265, 0.246]	0.909	[-0.568, 0.475]	No
JP	Yes	0.994	[0.979, 1.000]	Yes	-0.888	[-0.263, 0.259]	0.552	[-0.466, 0.375]	No

After establishing the MICOM, the PLS-MGA was conducted to check the moderating role of perceived organizational response to a crisis in the organizational identification - job performance link. The results in Table 6 show that perceived organizational response to a crisis significantly moderates the relationship between organizational identification and job performance, further supporting H6. Thus, at a low level of perceived organizational response to a crisis, the positive influence of organizational identification on job performance would be greater than at a high level of perceived organizational response to a crisis.

**Table 6:** Results of moderation analysis.

Relationship	Low-PORC	High- PORC	β Differences	Henseler's MGA Test (p-value)	Permutation Test (p-value)	Result
OI → JP	0.664∗∗∗	0.359∗∗∗	0.305	0.001	0.002	Yes

This study introduced four control factors, including demographic data (i.e., age, gender) and job-related variables (i.e., position, contract type), to assess their potential impact on job performance. However, none of these control variables affected job performance in the setting of the COVID-19 crisis (*t*-value was insignificant).

## 5 Discussion and implications

### 5.1 Discussion

The goal of this study was to examine the relationships between perceived socially responsible HRM, organizational identification, and job performance. The findings indicated that perceived socially responsible HRM had a beneficial effect on job performance. This result is congruent with the findings of Shen and Benson (2016) and Manzoor et al. (2019). Additionally, the results demonstrated that employees' perceptions of socially responsible HRM have a favorable effect on organizational identification. It was found that when employees perceive that their organization employs socially responsible HRM practices with good values and moral ethics, they feel more identified with their organization. This finding was in line with those of previous studies (Shen and Benson, 2016; Wang et al., 2017) that organizations’ socially responsible HRM practices could make employees feel proud to work for the organization, enhance their self-concept, and improve their identification with the organization. Moreover, this study found that organizational identification acts as a mediator in the relationship between perceived socially responsible HRM and job performance. This finding was aligned with the research results of Walumbwa et al. (2011) and Wang et al. (2017).

The finding also suggested that employees' job performance is positively influenced by their perception of the organization's response to a crisis. This finding was consistent with the results of Bardoel et al. (2014) and Vo-Thanh et al. (2020). Moreover, interestingly, the results demonstrated that organizational identification has a lower influence on the work performance of employees who perceive good organizational responses to a worldwide crisis compared to those who perceive poor organizational responses. In other words, the effect of organizational identification on job performance is stronger at a low level of perceived organizational response to a crisis than at a high level. This result supports the suggestion of Lian et al. (2022) on the role of the ways in which leaders of organizations respond to a crisis such as the COVID-19 pandemic and organizational identification with employee job performance.

### 5.2 Theoretical implications

This study provides several significant theoretical implications. First, this research provides information on the applicability of the social exchange theory and social identity theory in enhancing our knowledge of the effect of employees' perceptions of socially responsible HRM on their organizational identification and job performance during a crisis, such as the COVID-19 pandemic. The results demonstrated that perceived socially responsible HRM positively influences job performance. This result could be explained by the principle of social exchange theory (Blau, 1964) that an enterprise's socially responsible HRM may inspire employees to perceive that they are important members of the enterprise, leading to an underlying need to reciprocate by engaging in behaviors and activities that benefit the organization (Luu, 2021; Luu et al., 2022; Newman et al., 2016), which, in turn, promotes their job performance.

Moreover, the findings showed that employees’ perceived socially responsible HRM has a positive influence on organizational identification. This finding supported the social identity theory (Ashforth and Mael, 1989; Tajfel et al., 1979). Moreover, when organizations embrace socially responsible HRM initiatives that directly benefit their employees, these employees will develop higher degrees of organizational identification by reciprocating by engaging psychologically in the workplace. This result was aligned with those of previous research (He et al., 2014; Newman et al., 2016) and supported the principle of social exchange theory (Blau, 1964; Cropanzano and Mitchell, 2005).

Additionally, this research develops our knowledge of the mediating role of organizational identification in the nexus between perceived socially responsible HRM and job performance. The results indicated that employees’ perceived socially responsible HRM results in greater organizational identification, which, in turn, promotes job performance. In other words, organizational identification acted as a mediator in the association between socially responsible HRM and job performance. This finding further supported the principle of social exchange theory (Blau, 1964; Cropanzano and Mitchell, 2005) and the social identity theory (Ashforth and Mael, 1989; Tajfel et al., 1979).

Furthermore, this study contributes to our understanding of the impact of employees' perceptions of organizational response to a global crisis on their job performance and the moderating effects of perceptions of organizational reaction to a global crisis in the relationship between organizational identification and job performance. The finding indicated that perceived organizational response to a crisis positively influences employees’ job performance. This result was in line with those of Bardoel et al. (2014) and Vo-Thanh et al. (2020), further supporting the social exchange theory. Moreover, the results of this study demonstrated that perceived organizational response to a crisis negatively moderates the relationship between organizational identification and job performance. Our study contributes to the growing body of literature on crisis management by proposing that the ways in which leaders of organizations respond to a global crisis could interact with employees' organizational identification to change the outcomes that employees experience while they are working.

### 5.3 Practical implications

This study also highlights several significant practical implications. First, organizations should invest in socially responsible HRM since these practices are essential for the sustainable development of their organizations, and employees' perceived socially responsible HRM indirectly impacts their job performance via organizational identification. Organizations should build and implement all three aspects of socially responsible HRM: legal compliance HRM (ensuring equal opportunity in HRM; paying above minimum salary and basing compensation on performance, complying with regulations on hiring, contract labor, social insurance contributions and other compulsory social benefits, complying with the regulations regarding the working hours and overtime; eliminating child labor or forced labor; having systematic and comprehensive workplace health and safety regulations; appointing staff monitoring labor standards in business partners, for example, suppliers and contractors); employee-oriented HRM practices (adopting flexible working hours and employment programs achieving work–life balance; providing adequate training and development opportunities to employees) and workplace democracy practices (stimulating bottom-up voice in the organization; allowing employees to participate in decision-making and total quality management and their suggestions and ideas are appreciated by managers; trade unions can represent and safeguard employees' rights and participate in defining labor conditions); general CSR facilitation HRM (appointing adequate staff implementing general CSR initiatives toward shareholders, community, environment, employees, customers, and other partners; enforcing employee participation in CSR activities; rewarding employees who contribute to environmental protection, charity, communities, and other CSR initiatives; providing employment opportunities to all applicants in need and who are local). The organization should incorporate general CSR facilitation HRM into employee job performance appraisal and link CSR activities with employees’ work.

Furthermore, given that perceived organizational response to a crisis such as the COVID-19 pandemic positively influences job performance and moderates the relationship between organizational identification and job performance, enterprises should implement appropriate strategies to counter the crisis to promote employees' job performance. Managers should respond to a global crisis such as the COVID-19 pandemic with prompt and appropriate plans and scenarios and take care of employees' needs and difficulties resulting from the crisis. The organization's management board should also show that they do everything they can to respond to a global crisis and create a decent workplace for employees. These factors demonstrate the enterprise's CSR to employees while also assisting them in maintaining their job performance during a crisis. Employees who are satisfied with the enterprise's response to a crisis have more faith in the organization, feel that they identify more with the organization, and are willing to exert more effort to help the organization overcome challenges. Therefore, to maintain a competitive advantage and preserve positive employee attitudes and behaviors during and after a crisis, every organization must adopt an effective crisis response strategy.

Finally, the company should develop a recovery strategy for a crisis such as the COVID-19 pandemic that focuses on employees who have a low level of organizational identification to enhance their job performance during a global crisis. The reason for this is that the results of the current study demonstrated that organizational identification has a stronger influence on the work performance of employees who perceive poor organizational responses to a global crisis than those who perceive good organizational responses. Additionally, employees should be included in the process of the organizational response to a global crisis. Participation in a company's strategic planning can help employees feel more secure, identified, and engaged, which results in improving their job performance.

## 6 Limitations and suggestions for further study

Despite its substantial contributions, this study has a number of shortcomings. First, this study only used cross-sectional data, and although we presented the research in a way that proactively addresses concerns with CMB (Podsakoff et al., 2003), this type of data could still overlook the antecedent–consequent relationship between employees' perceived socially responsible HRM, organizational identification, perceived organizational response to a crisis, and job performance. Therefore, future research should conduct a longitudinal analysis with three stages of a survey for the independent variable, mediating and moderating variables, and dependent variable. Second, the study sample included full-time Vietnamese employees working in industries that have been more heavily impacted by COVID-19 than others (including trade, wholesale, and retail; manufacturing or processing; construction and real estate; tourism, restaurant, leisure, and hotel services; and transportation, warehousing, and logistics). Further research can be conducted to gain a better understanding of the distinctions among all industries in the economy. In addition, this study was conducted in Vietnam, a developing country; therefore, from the institutional and cultural perspectives, it is recommended that to gain more insights into the relationships between employees’ perceived socially responsible HRM and their organizational identification and job performance, future research should include other countries. Finally, this study relied solely on self-reported information from individual-level employee perspectives. Additional research from the perspective of top managers or at the organizational level could provide a full evaluation of the influence of socially responsible HRM on employee attitudes and behaviors.

## Declarations

### Author contribution statement

Thinh-Van Vu, Ph.D: Conceived and designed the experiments; Performed the experiments; Analyzed and interpreted the data; Contributed reagents, materials, analysis tools or data; Wrote the paper.

### Funding statement

This research did not receive any specific grant from funding agencies in the public, commercial, or not-for-profit sectors.

### Data availability statement

Data will be made available on request.

### Declaration of interest's statement

The authors declare no conflict of interest.

### Additional information

No additional information is available for this paper.
