# Supplementary material for: Perceived socially responsible HRM, employee organizational identification, and job performance: the moderating effect of perceived organizational response to a global crisis
Source: Heliyon. 2022 Nov 16;8(11):e11563. doi: 10.1016/j.heliyon.2022.e11563 (PMC9699979; doi:10.1016/j.heliyon.2022.e11563)
Supplement: Multimedia component 1 [file mmc1.docx]

**A SURVEY OF EMPLOYEES’ PERCEIVED Socially Responsible Human Resource Management, Organizational Identification, Perceived Organizational Response to a Crisis (covid-19), and Job Performance**

Dear Sir/Madam,

We are …

Currently, we are doing research on the employees’ perception of their organization’s Socially Responsible Human Resource Management and employees’ organizational identification and job performance during a crisis - the COVID-19 pandemic. The research results can give theoretical and practical implications for human resource management activities in the new and changing context. Thank you in advance for your time.

This survey investigates full-time employees (the top management level is excluded) working in the following industries in Vietnam:

1: Trade, wholesale, and retail

2: Transportation, warehousing, and logistics

3: Construction and real estate

4: Tourism, restaurant, leisure, and hotel services

5: Manufacturing or processing (e.g., textile, footwear, electronic, food processing)

***If you are not a full-time employee working in these industries, please skip this survey.***

--

**Confidentiality Pledges**

Participation in this survey is voluntary and entirely anonymous. The data collected will be confidential and will be used for scientific purposes only.

*1) I agree to participate in this survey.*

*2) Participation in the survey is completely voluntary.*

*3) I permit the researcher to use the responses. I understand that my identity will be kept confidential and will not be displayed on research results.*

--

**Notes** *1) In this survey, we do not distinguish the organization from the company. All of these terms indicate the place where you are working.*

*2) If you fill the questionnaire with your mobile phone, please rotate it horizontally to make it easier to read.*

*If you need any further queries, please send an e-mail to my address …*

--

**I. Are you currently working in one of the following industries?**

- *1: Trade, wholesale, and retail*
- *2: Manufacturing or processing (e.g. textile, footwear, electronic, food processing)*
- *3: Construction and real estate*
- *4: Tourism, restaurant, leisure, and hotel services*
- *5: Transportation, warehousing, and logistics*
- ***6. Others - Thank you! (If a respondent chooses this option, it automatically stops here)***

**II. Demographic Information**

2.1. Your age?

- ≤30
- 31-40
- ≥41

2.2. Your gender?

- Male
- Female
- Others

2.3. Your position in the organization?

- Non-managerial employee
- First-line manager
- Middle manager

2.4. What is the type of your work contract with the current organization?

- A contract under 1 year
- A contract from 1-2 years
- A contract from 2-3 years
- Indefinite contract

2.5. Size of your organization in terms of number of employees?

- <50
- 51-100
- 101-200
- 201-500
- 501-1000
- 1001-2000
- >2000

**III. Please answer the questions about the main content of the survey below.**

***Note: Use the scale and tick the number that best describes your opinion as follows:* (1: Totally disagree 🡪 5: Totally agree)**

| **Scales’ items** | **Response options (1: Totally disagree 🡪 5: Totally agree)** | | | | |
| --- | --- | --- | --- | --- | --- |
| **Perceived Socially Responsible HRM** | | | | | |
| ***To what extent do you agree with the following statements related to “Your Organization’s Legal Compliance HRM”?*** | | | | | |
| - My organization ensures equal opportunity for employees in HRM | 1 | 2 | 3 | 4 | 5 |
| - Employees in my organization are paid above minimum wages and based on their performance | 1 | 2 | 3 | 4 | 5 |
| - My organization complies with the regulations regarding the contract labor, working hours, and compulsory social benefits | 1 | 2 | 3 | 4 | 5 |
| - My organization does not employ child labor or forced labor | 1 | 2 | 3 | 4 | 5 |
| - My organization has clear and detailed regulations on occupational health and safety | 1 | 2 | 3 | 4 | 5 |
| - My organization appoints staff monitoring labor standards in business partners; for example, suppliers and contractors | 1 | 2 | 3 | 4 | 5 |
| ***To what extent do you agree with the following statements related to “Your Organization’ Perceived Employee-oriented HRM”?*** | | | | | |
| - My organization adopts flexible working hours and employment programs achieving work-life balance | 1 | 2 | 3 | 4 | 5 |
| - My organization provides adequate training and development opportunities to employees | 1 | 2 | 3 | 4 | 5 |
| - Bottom-up voice is stimulated in the organization | 1 | 2 | 3 | 4 | 5 |
| - Employees are allowed to participate in decision making and total quality management; and their suggestions and ideas are appreciated by managers | 1 | 2 | 3 | 4 | 5 |
| - Unions can represent and protect workers’ rights and can be involved in determining labor terms | 1 | 2 | 3 | 4 | 5 |
| ***To what extent do you agree with the following statements related to “Your Organization’ General CSR Facilitation HRM”?*** | | | | | |
| - My organization appoints adequate staff implementing general CSR initiatives (toward shareholders, community, environment, employees, customer, and other partners) | 1 | 2 | 3 | 4 | 5 |
| - My organization enforces employee participating on CSR activities; rewards employees who contribute to environmental protection, charity, communities, and other CSR activities | 1 | 2 | 3 | 4 | 5 |
| - My organization gives equal opportunity employment to all candidates, including those who are in difficulty and who are local | 1 | 2 | 3 | 4 | 5 |
| **To what extent do you agree with the following statements related to “Your Organizational Identification”?** | | | | | |
| - When someone criticizes the organization, it feels like a personal insult | 1 | 2 | 3 | 4 | 5 |
| - I am very interested in what others think about the organization | 1 | 2 | 3 | 4 | 5 |
| - When I talk about the organization, I usually say ‘we’ rather than ‘they’ | 1 | 2 | 3 | 4 | 5 |
| - The organization’s successes are my successes | 1 | 2 | 3 | 4 | 5 |
| - When someone praises the organization, it feels like a personal compliment | 1 | 2 | 3 | 4 | 5 |
| **To what extent do you agree with the following statements related to “Organizational Response to a Crisis** **of COVID-19” in your enterprise?** | | | | | |
| - I am satisfied with the way that my employer responded to crisis by prompt and appropriate plans and scenarios | 1 | 2 | 3 | 4 | 5 |
| - I am satisfied that my organization’s management board did everything that it could have in response to crisis | 1 | 2 | 3 | 4 | 5 |
| - I am satisfied with the way that my organization’s management board took care of its employees’ needs and difficulties resulting from crisis | 1 | 2 | 3 | 4 | 5 |
| **To what extent do you agree with the following statements related to “Your Job Performance”?** | | | | | |
| - I fulfill my job responsibilities | 1 | 2 | 3 | 4 | 5 |
| - I meet the performance standards and expectations of the job | 1 | 2 | 3 | 4 | 5 |
| - My performance level satisfies my manager | 1 | 2 | 3 | 4 | 5 |
| - I perform better than many other ones who perform the same job | 1 | 2 | 3 | 4 | 5 |
| - I have adequate competencies to carry out my work effectively | 1 | 2 | 3 | 4 | 5 |
| - I produce high-quality work | 1 | 2 | 3 | 4 | 5 |
| - I fulfill my job responsibilities | 1 | 2 | 3 | 4 | 5 |

**Thank you very much for your response!**
